# Evaluation of Changes in Morphology and Function of Human Induced Pluripotent Stem Cell Derived Cardiomyocytes (HiPSC-CMs) Cultured on an Aligned-Nanofiber Cardiac Patch

**DOI:** 10.1371/journal.pone.0126338

**Published:** 2015-05-19

**Authors:** Mahmood Khan, Yanyi Xu, Serena Hua, Jed Johnson, Andriy Belevych, Paul M. L. Janssen, Sandor Gyorke, Jianjun Guan, Mark G. Angelos

**Affiliations:** 1 Department of Emergency Medicine, Davis Heart Lung Research Institute, The Ohio State University Wexner Medical Center, Columbus, OH, United States of America; 2 Department of Materials Science and Engineering, Davis Heart Lung Research Institute, Ohio State University, Columbus, OH, United States of America; 3 Nanofiber Solutions, Columbus, OH, United States of America; 4 Department of Physiology and Cell Biology, Davis Heart Lung Research Institute, The Ohio State University Wexner Medical Center, Columbus, OH, United States of America; Centro Cardiologico Monzino, ITALY

## Abstract

**Introduction:**

Dilated cardiomyopathy is a major cause of progressive heart failure. Utilization of stem cell therapy offers a potential means of regenerating viable cardiac tissue. However, a major obstacle to stem cell therapy is the delivery and survival of implanted stem cells in the ischemic heart. To address this issue, we have developed a biomimetic aligned nanofibrous cardiac patch and characterized the alignment and function of human inducible pluripotent stem cell derived cardiomyocytes (hiPSC-CMs) cultured on this cardiac patch. This hiPSC-CMs seeded patch was compared with hiPSC-CMs cultured on standard flat cell culture plates.

**Methods:**

hiPSC-CMs were cultured on; 1) a highly aligned polylactide-co-glycolide (PLGA) nanofiber scaffold (~50 microns thick) and 2) on a standard flat culture plate. Scanning electron microscopy (SEM) was used to determine alignment of PLGA nanofibers and orientation of the cells on the respective surfaces. Analysis of gap junctions (Connexin-43) was performed by confocal imaging in both the groups. Calcium cycling and patch-clamp technique were performed to measure calcium transients and electrical coupling properties of cardiomyocytes.

**Results:**

SEM demonstrated >90% alignment of the nanofibers in the patch which is similar to the extracellular matrix of decellularized rat myocardium. Confocal imaging of the cardiomyocytes demonstrated symmetrical alignment in the same direction on the aligned nanofiber patch in sharp contrast to the random appearance of cardiomyocytes cultured on a tissue culture plate. The hiPSC-CMs cultured on aligned nanofiber cardiac patches showed more efficient calcium cycling compared with cells cultured on standard flat surface culture plates. Quantification of mRNA with qRT-PCR confirmed that these cardiomyocytes expressed α-actinin, troponin-T and connexin-43 *in-vitro*.

**Conclusions:**

Overall, our results demonstrated changes in morphology and function of human induced pluripotent derived cardiomyocytes cultured in an anisotropic environment created by an aligned nanofiber patch. In this environment, these cells better approximate normal cardiac tissue compared with cells cultured on flat surface and can serve as the basis for bioengineering of an implantable cardiac patch.

## Introduction

Heart failure is a growing epidemic without a known cure. Once diagnosed, the disease course is generally progressive and non-reversible with a 5-year survival rate of about 50%, resulting in approximately 300,000 deaths per year in the US [1]. Ischemic cardiomyopathy is a principal cause of heart failure, frequently following myocardial infarction with resultant remodeling of the left ventricle (LV) resulting in dilation, fibrosis and subsequent reduced ejection fraction and cardiac output. Current clinical therapy (other than heart transplantation), is palliative and fails to reverse the functional cardiomyocyte loss due to post-ischemic remodeling.

Stem cell based therapies, with their myocardial regeneration potential, offers a different therapeutic paradigm. Despite a number of pre-clinical and early clinical studies utilizing stem cell therapy, there remain significant questions regarding delivery, survival and effects of stem cell based therapy in the heart [2,3]. The most common methods of stem cell delivery to the heart have been intravenous, intracoronary and direct intramyocardial injections. These methods are relatively inefficient due to dispersion of cells and cell loss. A recent clinical study reported 2.6 ± 0.3% early retention of stem cells in the heart after intracoronary administration compared with 11 ± 3% cell retention following intramyocardial injection [4]. Overall, cell retention is limited with > 90% of injected cells disappearing in the first few days [5]. Four weeks after injection, <2% of cells are found. Cell loss and retention is in large part due to the hostile ischemic microenvironment present in the scarred, fibrotic myocardium [5].

Combining stem cell therapy with optimal scaffolding derived from natural or synthetic polymers to form a cardiac “patch” may allow for regeneration and repair of injured or damaged regions of the heart. For tissue engineering, using biodegradable scaffolds combined with stem cell therapy is an alternative strategy to cell infusion or injection and may provide a repository for cell delivery leading to improved early cell survival. We have developed a biodegradable scaffolding material which replicates the extracellular matrix of the heart for the purpose of providing anisotropic support for cardiomyocytes. While a number of stem cell types are available, this work focuses on the use of fully differentiated cardiomyocytes derived from human induced pluripotent stem-cells (hiPSCs). Utilization of induced pluripotent stem-cells present a number of advantages including reduced or absent need for immunosuppressive therapy due to the autologous nature of the cells when derived from the patient. The purpose of the current study is to compare structure, alignment, contractile function, electrical properties and mRNA expression of hiPSC-CMs cultured on an aligned nanofiber scaffold when compared to the cells cultured on flat cell culture plates. To better characterize the hiPSC-CMs, we have compared them to non-failing human heart tissues.

## Materials and Methods

### Scaffold Fabrication

Polymer nanofiber precursor solutions were manufactured (Nanofiber Solutions, Columbus, Ohio) by utilizing a FDA approved biodegradable polylactide-co-glycolide (PLGA). Eight wt% PLGA (PURAC, Netherlands) was dissolved in 1,1,1,3,3,3-hexafluoroisopropanol (Oakwood Chemicals) by heating the solution to 60°C followed by continuous stirring until the PLGA was completely dissolved. After cooling to room temperature, the solution was placed in a 60cc syringe with a 20 gauge blunt tip needle (Engineering Fluid Dispensing). The nanofibers were deposited to a thickness of approximately 50 microns onto a custom manufactured rotating wheel with a surface velocity of 15 m/s [6] by electrospinning using a high voltage DC power supply (Gamma High Voltage) set to +14kV, a 15 cm tip-to-substrate distance, and a 5 mL/hr flow rate. Average fiber diameter was 950 microns [7]. All scaffolds were placed in a vacuum overnight to ensure removal of residual solvent (typically less than 10 ppm) [8]. The aligned fiber scaffolds were then sterilized with 350 μJ/cm^2^ of UV radiation.

### Culturing and maintenance of hiPSC-CMs

Human iPSC-CMs were obtained from Cellular Dynamics International (CDI, Madison, WI) and were seeded onto either standard flat tissue culture plates or aligned nanofiber coated plates (Nanofiber Solutions, Columbus, OH) at a cell concentration of 8x10^5^ cells per 9.5cm^2^. Prior to the cell culturing, both plates were coated with 1% gelatin to aid cell attachment prior to seeding. HiPSC-CMs were seeded onto the gelatin coated plates and the cell culture media was replaced with maintenance media at 48 hours after plating as published earlier [9]. Cells were collected for further analysis at one-week post plating.

### Alignment of hiPSC-CMs seeded on nanofibers

The alignment of hiPSC-CMs seeded on flat or aligned nanofiber coated plates was characterized by cell anisotropic index (CAI). Samples were rinsed with PBS, treated with 4% of paraformaldehyde, permeabilized with 0.1% of Triton X-100 and stained with alexa flour 555 phalloidin (Invtrogen, Oregon) in sequence. Representative images were then taken by Olympus FV1000-Filter Confocal microscopy. These images were processed by the Welch window method to remove the edge effect first. A fast Fourier transformation algorithm was then applied to obtain the power spectrum pattern from the windowed image. An intensity-orientation histogram plot was then calculated based on the power spectrum. CAI value was defined as the possibility of cells aligned within ± 20 degrees of the principle aligned axis normalized by that of a purely random sample. Thus the CAI of a completely round cell is 0 and a higher CAI means a higher degree of cell alignment.

### Confocal imaging of cardiac markers in hiPSC-CMs *in vitro*

Immunofluorescence staining was performed in cells fixed with paraformaldehyde. The fixed cells were washed with PBS and then incubated with 2% goat serum and 5% bovine-serum albumin in PBS to reduce nonspecific binding. The cells or cardiac sections were then incubated for 2 hours at room temperature with mouse, anti-α-sarcomeric actinin (1:500, Sigma-Aldrich, MO), and connexin-43 monoclonal antibodies (1:500, Cell Signaling, MA). The sections were then incubated with the appropriate goat or rabbit anti-mouse secondary antibodies (1:1000) conjugated to Texas red and FITC. The nuclei were counterstained with HardSet mounting medium with DAPI (Vector Labs). The cells were visualized by Olympus FV1000 spectral confocal microscopy. Separate cells were also stained without primary antibodies to identify nonspecific binding.

### Mitochondrial staining of adherent hiPSC-CMs

To label the mitochondria, hiPSC-CMs were incubated with MitoTracker (Molecular Probes Inc, Oregon), which passively diffuse across the plasma membrane and accumulate in active mitochondria. Briefly hiPSC-CMs were cultured on coverslips inside a Petri dish filled with the culture medium. When cells reached the desired confluency, the media from the dish was replaced with pre-warmed (37°C) media containing staining solution of MitoTracker probe (200 nM). The cells were then incubated for 30 minutes with the Mitotracker containing media. After 30 minutes of staining, the cell culture media was replaced with fresh pre-warmed media and the cells were observed under the Zeiss Axiovert 135 fluorescence microscope and images were captured.

### Scanning Electron Microscopy (SEM) studies in hiPSC-CMs

The morphology of the hiPSC-derived cardiomyocyte monolayer on the cardiac patch and standard tissue culture plate were determined using scanning electron microscopy (SEM). The samples were prepared following previous reports [10,11]. Briefly, the patches were fixed with 4% paraformaldehyde for 1 hour. After rinsing with de-ionized water, they were dehydrated by 50%, 70%, 80%, 95% and 100% ethanol solutions in sequence. Hexamethyldisilazane was then used to chemically dry the samples. The samples were finally sputter-coated with gold and imaged with a FEI NOVA nanoSEM.

### Transmission Electron Microscopic (TEM) studies in hiPSC-CMs

Human iPSC-derived cardiomyocyte monolayers were grown on Permanox Chamber slides (Lab Tek), and hiPSC-CMs were cultured on aligned nanofiber patches. The cells were then fixed in 2.5% glutaraldehyde in 0.1M phosphate buffer with 0.1M sucrose pH = 7.4 for 1 hour. They were then post fixed in 1% osmium tetroxide for 30 minutes, en bloc stained in 2% uranyl acetate for 30 minutes and dehydrated in a graded series of ethanol. HPMA (hydroxy-propyl methacrylate) was used as an intermediate between ethanol and Eponate12 (Ted Pella Inc.) epoxy resin. Final embedding was in Eponate12 in inverted Beem (Ted Pella Inc.) capsules and polymerized overnight at 70°C. Capsules with cells were trimmed and 70 nm thick sections were obtained using a Leica EM UC6 ultra-microtome and a diamond knife. Observations were made using a FEI Spirit TEM at 80 kV and images captured with an AMT camera.

### Measurement of Intracellular Calcium (Ca^2+^) cycling

Intracellular Ca^2+^ cycling was recorded in hiPSC-CMs as described previously [12,13]. Briefly, hiPSC-CMs were loaded with 9 μmol/L Fluo-4 AM for 25 min at room temperature. 20–60 minutes was allowed for de-esterification. Cells were then perfused with the following external solution (mmol/L): 140 NaCl, 5.4 KCl, 2.0 CaCl_2_, 0.5 MgCl_2_, 10 HEPES, and 5.6 glucose (pH 7.4). To induce Ca^2+^ transients, cells were paced with extracellular platinum electrodes. Intracellular Ca^2+^ imaging was performed using line-scan mode of Nikon A1R confocal microscope. The fluorescent probe was excited with the 488 nm (argon laser) and emission was collected at 500–550 nm. The fluorescent signal was expressed as F/F_0_, where F is the fluorescence at time t and F_0_ represents the background signal.

### Analysis of gap junctions (Connexin-43) by confocal microscopy

Connexin-43 (CX-43) expression of seeded cells was quantitatively analyzed, as previously described [14], by creating a mask of cell borders based on actin staining followed by measurement of CX-43 staining within the mask using ImageJ analysis software. The mask was generated using the pencil tool with 20 pixel line width by tracing over cell boundaries according to the actin staining. Positive CX-43 staining was defined as pixels with gray values between 128 and 255. CX-43 expression per cell was then calculated as the total number of positive CX-43 pixels divided by the total cell area.

### Measurement of action potential recordings

#### i. Patch-clamp method

Action potentials (APs) from single hiPSC-CMs were recorded using whole-cell patch clamp method with Axopatch 200B amplifier (Molecular Devices, CA). External solution contained (mM): 140 NaCl, 5.4 KCl, 2.0 CaCl_2_, 0.5 MgCl_2_, 10 HEPES, and 5.6 glucose (pH 7.4). Patch pipettes were filled with a solution that contained (mM): 90 K-aspartate, 50 KCl, 5 MgATP, 10 NaCl, 1 MgCl2, 0.1 Tris GTP, 10 HEPES (pH 7.2). APs were evoked with 4 ms depolarizing current pulse with amplitude 50% above the threshold.

#### ii. Optical recordings

To record electrical properties from multicellular preparations of hiPSC-CMs, cells were loaded with voltage-sensitive dye di-4-AN(F)EPPTEA (3 μg/ml, 10–15 min at room temperature) [15]. The dye was excited with 488 nm line of argon laser, and fluorescence was collected using 500–550 nm and 663–738 nm emission filters. The signal was acquired using resonant scanner of Nikon AR1 confocal microscope at 420 frames (512 x 32 pixels) per second acquisition rate. The pixel size was 0.41 μm. The ratio of green to red fluorescent signals was calculated and the resulting signal was expressed as (F-F_0_)/F_0_, where F is the fluorescence at time t and F_0_ represents the background signal.

### Measurement of cardiac genes by quantitative real-time transcription polymerase chain reaction (qRT-PCR)

Both hiPSC-CMs and human cardiac tissues were mixed with Trizol for total RNA extraction. cDNA was synthesized using High Capacity cDNA Reverse Transcriptase kit [Applied Biosciences 4368814) according to manufacturer’s instructions. RT-PCR with GJA1 (Connexin-43), TNNC1 (Troponin C), ACTN2 (Alpha actinin), and GAPDH primers (Table 1) were performed using Fast SyberGreen Master Mix [Applied Biosciences 4385610].

**Table 1:** Primer list.

Primer	Forward Sequence	Reverse sequence
GJA1	CTGAGTGCCTGAACTTGCCT	CTGGGCACCACTCTTTTGC
ACTN2	GTGAACACCCCTAAACCCGA	ATCCTGTTAGCCGCTGTCTC
TNNC2	GCCCAATGGAGGAGTCCAAA	CAAAGTGAGCCTCAATCAGCG
GAPDH	GAAGACGGGCGGAGAGAAA	GAAGACGGGCGGAGAGAAA

Non-failing human heart tissues were collected and processed from non-transplantable donor hearts. Immediately after the heart was removed from the donor, it was flushed through the coronary arteries with cold cardioplegic solution, and transported to the lab. Left ventricular tissue samples were immediately frozen in liquid nitrogen, and stored at -80 Celsius.

### Ethics Statement

Human cardiac tissue was obtained from the Lifeline of Ohio Organ Procurement program (http://lifelineofohio.org). Procurement and use of these tissues was reviewed by the Institutional Review Board of The Ohio State University and a waiver of consent was granted for this study. These tissues were used according to the Ohio State University guidelines regarding the use of data and/or specimens.

## Results

### Scaffold mechanical characterization

Five tensile dogbones were cut from aligned PLGA nanofiber sheets according to the American Society for Testing and Materials (ASTM) D638 Type V and as described previously [16]. Tensile testing was performed using an MTS Systems Corporation load frame with a 25lb. load cell and an elongation rate of 50mm/min. The ultimate tensile strength (UTS) was 12.33 MPa, the elongation to failure was 251.4% and the Young’s modulus was 87.9 MPa. The Young’s modulus is slightly higher than published values [17,18], but acceptable for a tissue engineered cardiac patch. The results are shown in Table 2.

**Table 2:** Mechanical properties for aligned PLGA nanofiber scaffolds.

Sample	UTS (MPa)	% Elongation	Modulus (MPa)
1	13.87	277.7	99.2
2	12.20	288.6	85.1
3	9.691	190.3	70.5
4	11.19	277.7	123.6
5	14.71	223.0	60.9
**Mean**	12.33	251.4	87.9
**SD**	2.019	42.74	24.7

Ultimate tensile strength (UTS) and Young’s modulus are recorded in megaPascals (MPa) with the standard deviation (SD) shown.

### Improved alignment of hiPSC-CMs on nanofibers

**Fig 1:**
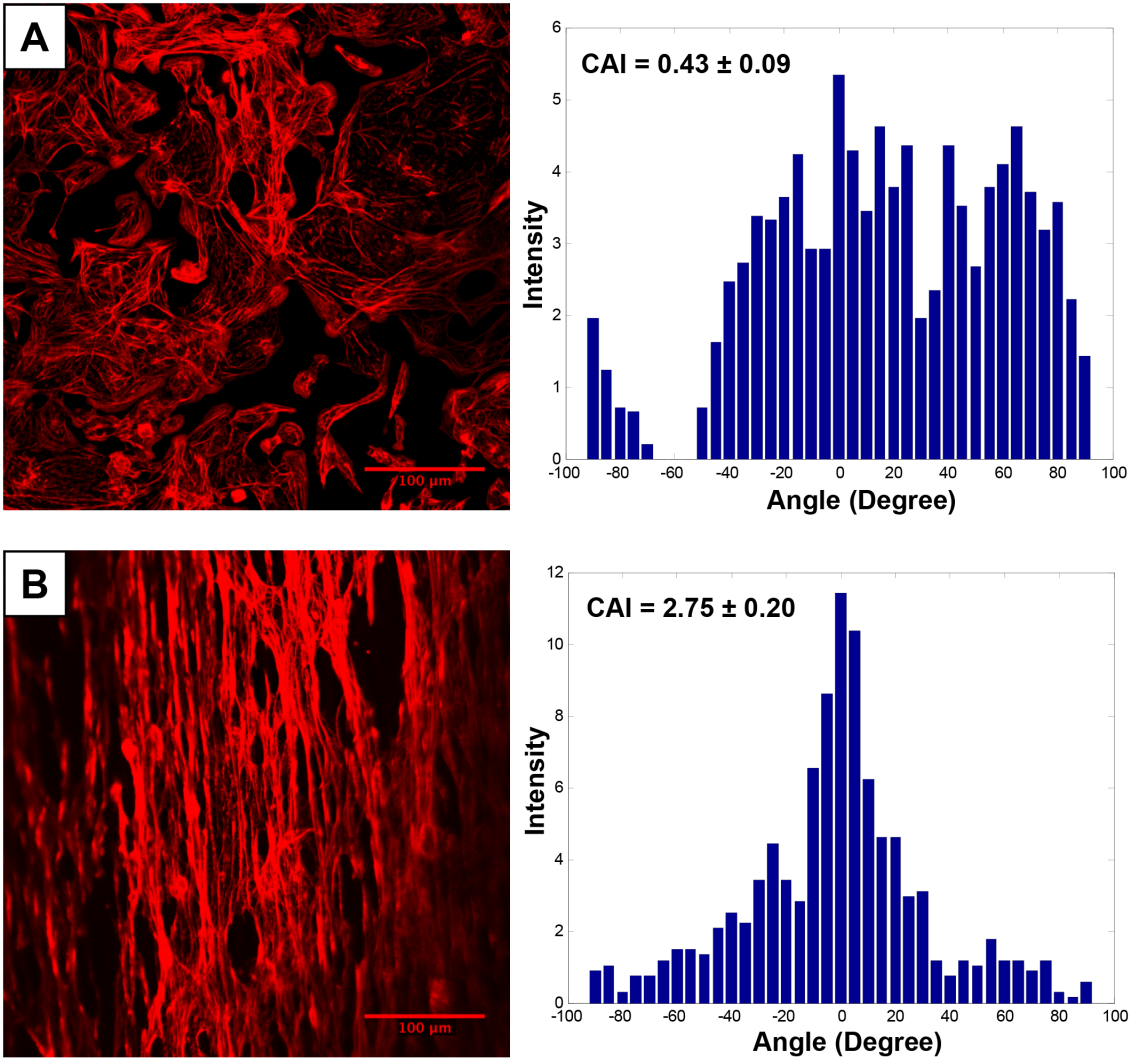
The alignment of hiPSC-CMs. Cells seeded on **A)** Flat surface plate and **B)** Aligned nanofiber characterized by cell anisotropic index (CAI). The hiPSC-CMs were stained with phalloidin dye (Red) to determine the alignment of seeded cells. CAI was significantly increased in the aligned nanofiber group.

When cultured on flat plates, hiPSC-CMs were evenly distributed and assumed a round morphology (Fig 1A). No obvious cell alignment was observed (CAI = 0.43 ± 0.09). However, when cultured on aligned nanofibers after 7 days cell morphology was changed. Cells were elongated and cell alignment (CAI = 2.75 ± 0.20) was significantly increased compared with those on the flat surface plates (Fig 1B).

### Analysis of gap junctions (Connexin-43) in hiPSC-CMs by confocal microscopy

**Fig 2:**
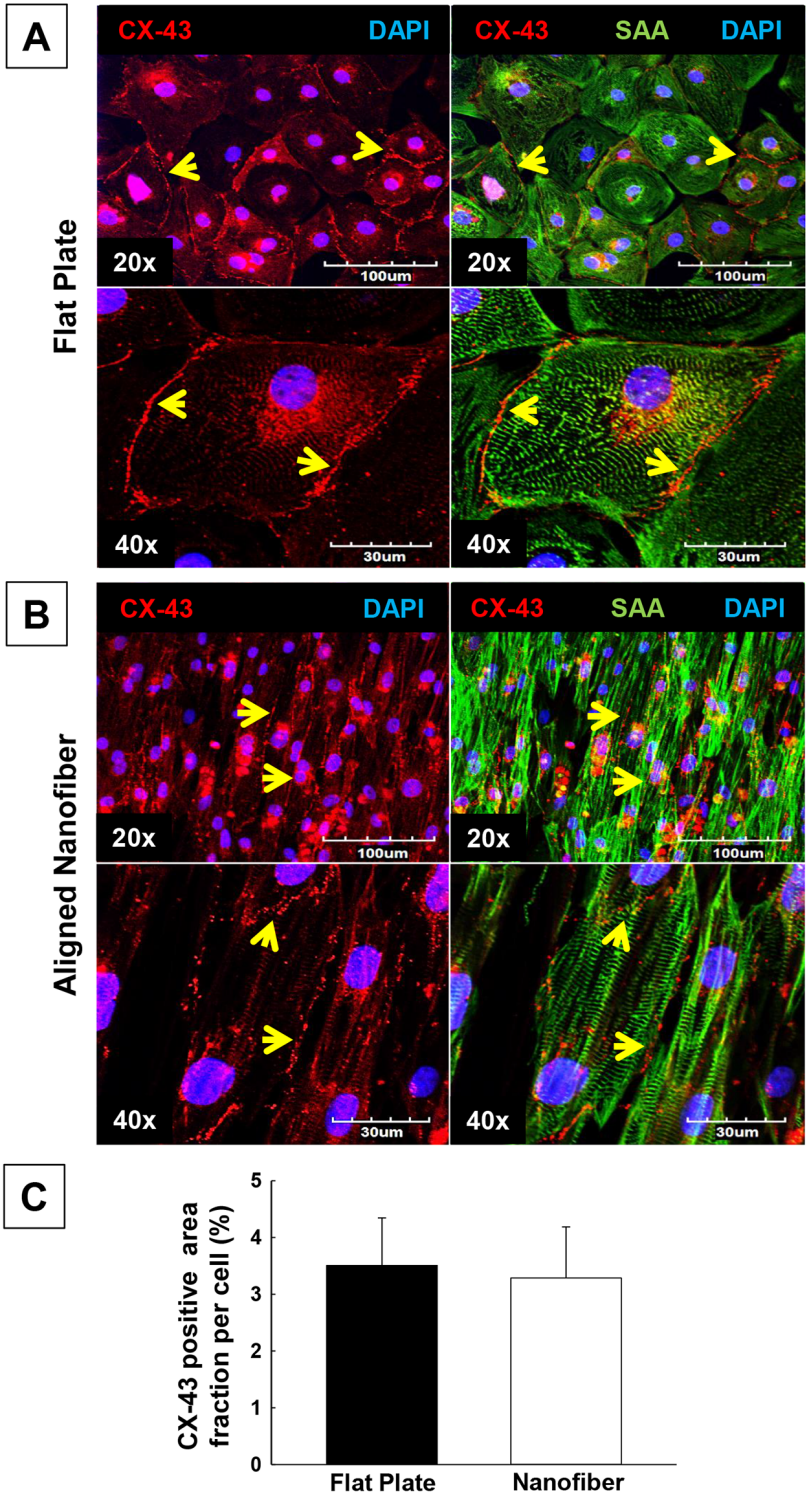
Analysis of gap junctions in hiPSC-CMs by confocal microscopy in cells seeded on flat plate and aligned nanofiber. The gap junctions in hiPSC-CMs was evaluated by CX-43 immunostaining of human cardiomyocytes seeded on flat surface versus aligned-nanofiber coated coverslips. **A**) Flat plate, CX-43 (Red), SAA (Green) and DAPI (Blue); **B**) Aligned nanofiber, CX-43 (Red), SAA (Green) and DAPI (Blue); **C**) Quantification of gap junctions (% CX-43 positive area fraction per cell) in hiPSC-CMs seeded on flat plate and aligned nanofiber. There were no significant differences between the two groups. All values expressed as mean ± SD (n = 4/group).

Double immunolabeling of connexin-43 (CX-43) and sarcomeric alpha actinin (SAA) demonstrated the evidence for electrical coupling by immunostaining of Connexin 43 (CX-43), which is a gap junctional protein responsible for cell-cell electrical coupling (Fig 2A and 2B). Our results showed that hiPSC-CMs seeded on flat surface and nanofibers demonstrated similar CX-43 expressions (3.51% ± 0.84% and 3.29% ± 0.90%, respectively, p<0.05, n = 4) (Fig 2C). These results indicated that in both groups, hiPSC-CMs can communicate well with each other and contribute to the synchronized functioning. These results further conclude that CX-43 was expressed in between cell-cell junctions showing evidence of electrical properties in hiPSC-CMs cultured on aligned nanofiber.

### Mitochondrial staining and alignment of hiPSC-CMs

**Fig 3:**
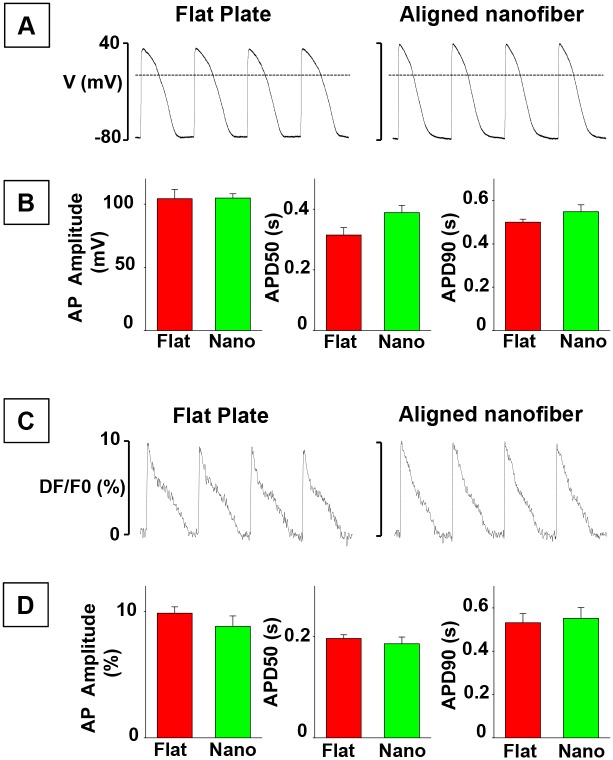
Confocal Imaging of mitochondria. Mito-tracker red staining shows alignment of Human iPSC-Cardiomyocytes seeded on **A)** Flat surface vs **B)** Aligned-nanofiber coated coverslips (32x & 200x). **C)** TEM imaging showing comparison of mitochondrial morphology and arrangement of hiPSC-CMs seeded on flat plate versus aligned nanofiber groups.

The mitochondrial staining of hiPSC-CMs with Mitotracker demonstrated mitochondrial arrangement in both flat surface (Fig 3A) and aligned nanofiber culture plates. The mitochondria in the aligned nanofiber group were oriented in the direction of the nanofibers (Fig 3B). In addition to the alignment of mitochondria in cardiomyocytes cultured on the nanofiber patch, these mitochondria as well as the extra-mitochondrial tissue appear to have a more mature appearance based on the increased density of cristae and the early sarcomere formation was observed (Fig 3C).

### Scanning Electron Microscopy of hiPSC-CMs

**Fig 4:**
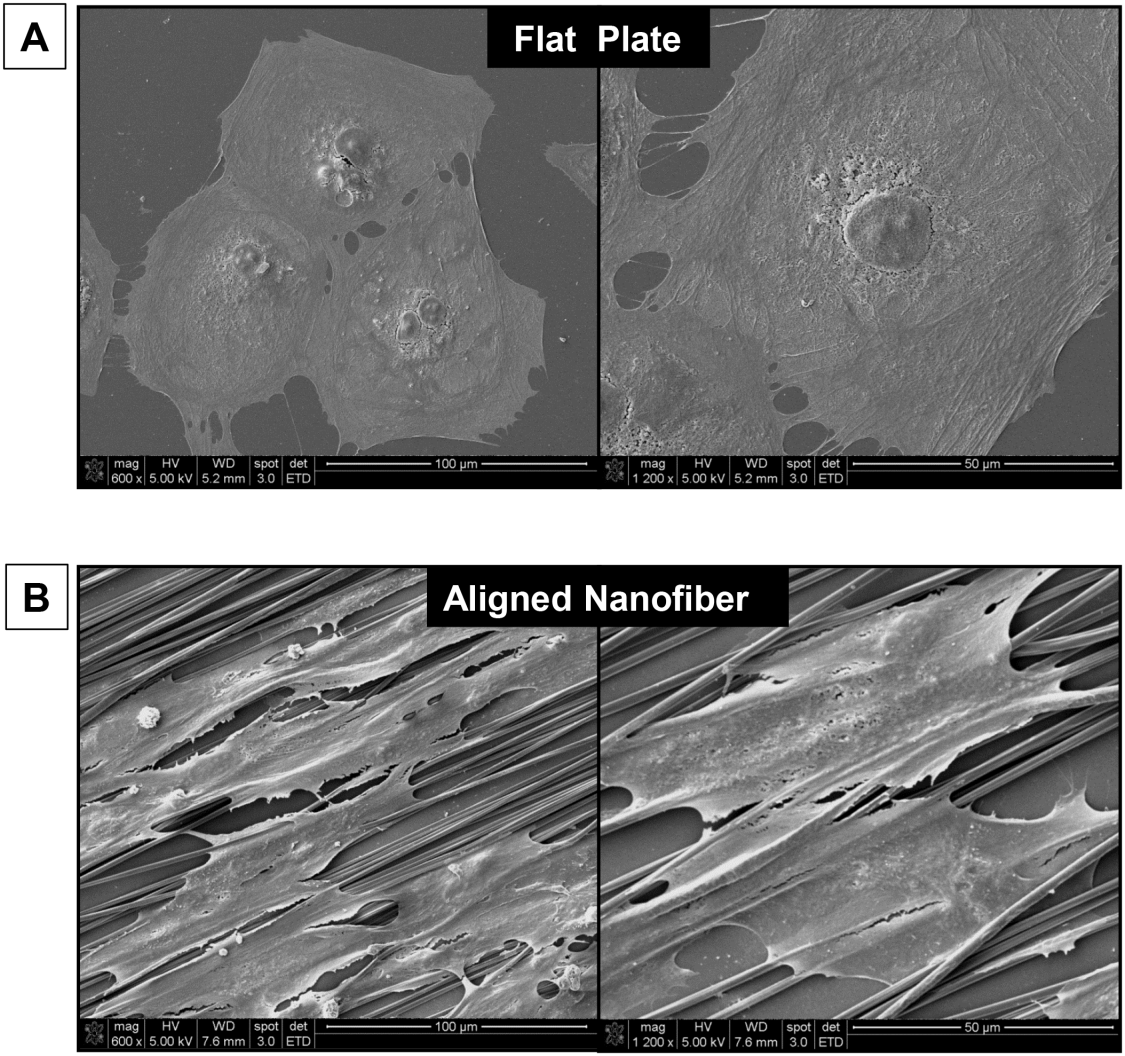
SEM imaging of Human iPSC-Cardiomyocytes. Cells seeded on **A)** Flat surface vs **B)** Aligned-nanofiber coated coverslips (600x & 1200x) show different cell morphology.

SEM images demonstrated >90% alignment of the nanofibers of the patch, which is similar to the natural alignment structure of the extracellular matrix of decellularized rat myocardium. In contrast with the random-distributed round shaped hiPSC-CMs on flat tissue culture plates (Fig 4A), the hiPSC-CMs seeded on aligned nanofibers exhibited stretched elongated morphology along aligned fiber orientation, demonstrating similar cell shape and alignment of the natural cardiomyocytes in vivo (Fig 4B).

### Transmission Electron Microscopy of hiPSC-CMs

**Fig 5:**
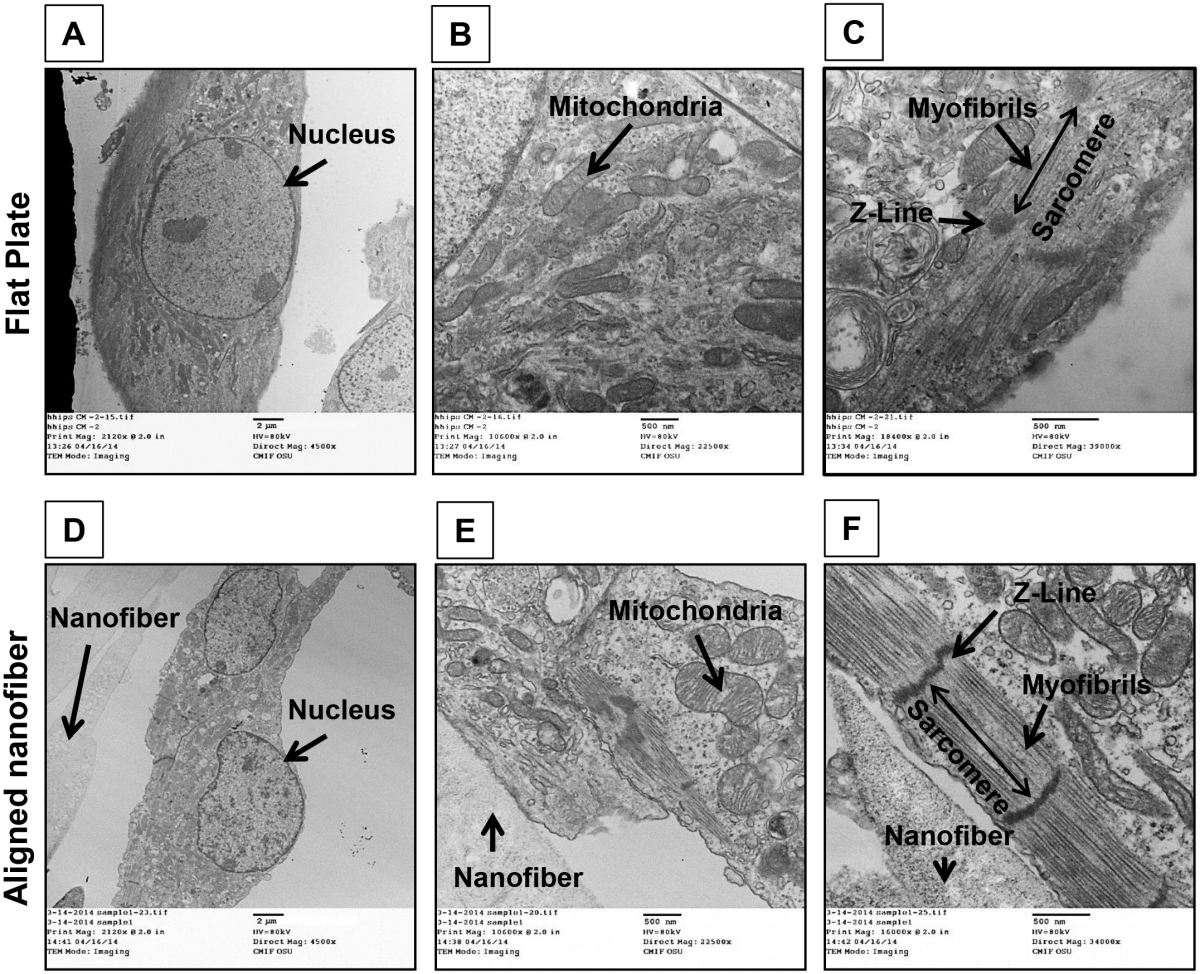
TEM imaging of Human iPSC-Cardiomyocytes. Cells seeded on Flat surface **A)** nucleus, **B)** mitochondria, **C)** myofibrils into sarcomere. Cells seeded on aligned nanofiber **D)** nucleus, **E)** mitochondria and **F)** myofibrils, (n = 4; 4500x, 22500x & 34000x) demonstrated myofibrils well-organized into sarcomeres with clear aligned Z-discs.

The aligned hiPSC-CMs in the patch had a sarcomere length of ~1.68 μm, which more closely approximates the fully matured adult sarcomere (~2.1 μm) [19] than that of hiPSC-CMs cultured on flat culture plates (~1.59 μm, Fig 5C and 5F). In addition, hiPSC-CMs on aligned nanofiber assembled into well-organized myofibrils with clear aligned Z-discs (Fig 5F) in contrast to those cultured on the flat culture plate (Fig 5C). These results demonstrate that hiPSC-CMs cultured on aligned nanofiber cardiac patches are more mature than those cultured on flat plates.

### Intracellular calcium (Ca^2+^) cycling in hiPSC-CMs

**Fig 6:**
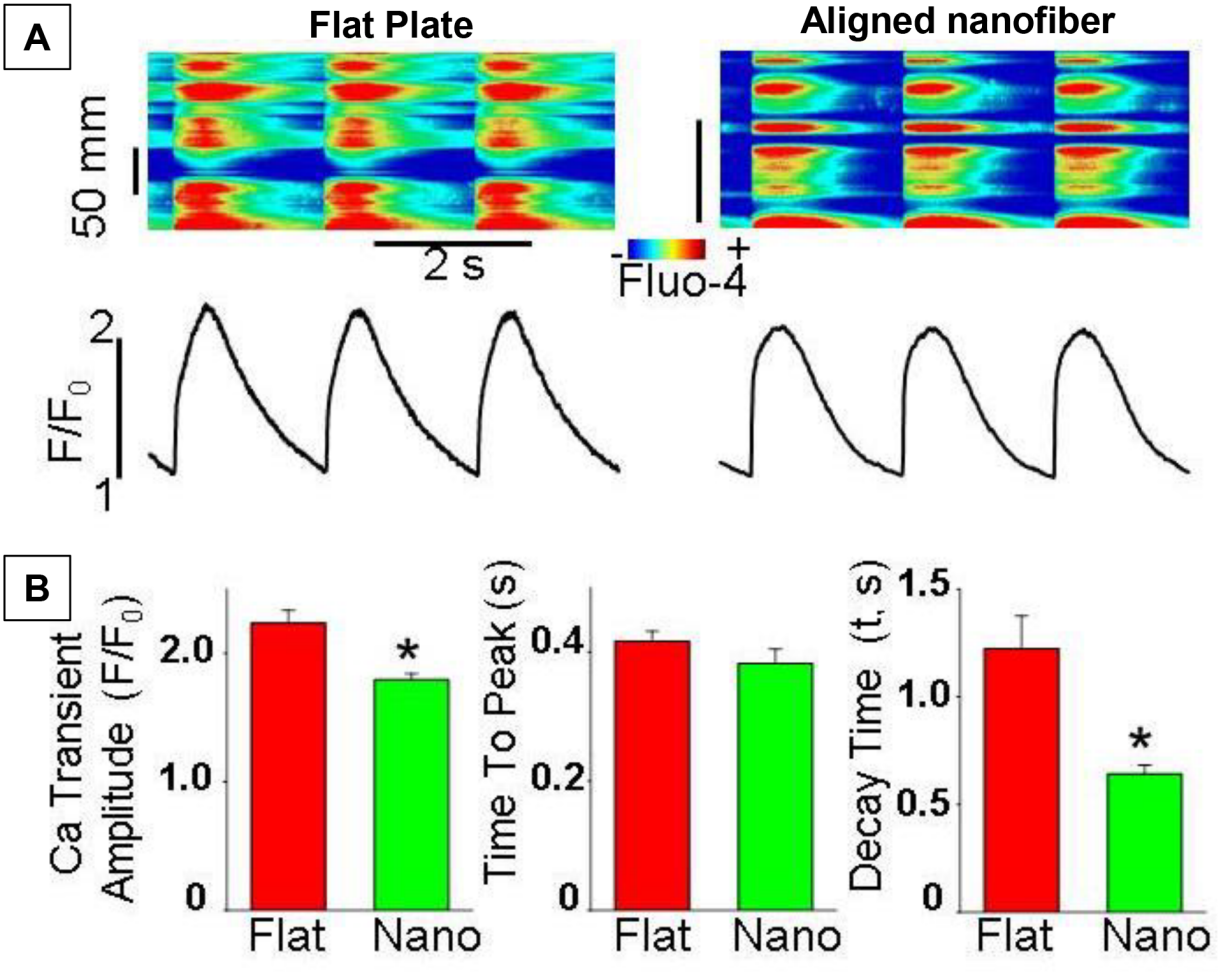
Intracellular calcium cycling in hiPSC-CMs. **A)** Line-scan images and temporal profiles of Fluo-4 fluorescence of hiPSC-CMs seeded on flat plate or on aligned nanofiber-coated coverslips. Ca^+2^ transients were evoked by electrical field stimulation at 0.5 Hz. **B)** Summary graphs showing average data for Ca^+2^ transient amplitude, time to peak and exponential decay constant (τ) of Ca^+2^ transients recorded in cells from flat plate (Flat, n = 10) and aligned nanofiber (Nano, n = 14) groups, respectively. *P<0.05 vs standard flat plate group. HiPSC-CMs cultured on aligned nanofibers have faster calcium cycling rate and higher contraction frequency than cells maintained on a flat surface.

We recorded Ca^+2^ transients induced by field stimulation in hiPSC-CMs cultured on standard flat culture plates and on aligned nanofibers (Fig 6A). The amplitude of Ca^+2^ transients exhibited a small but significant decrease in the nanofiber group compared to the flat bottom group. Whereas the time to peak values were similar for both groups, the decay of Ca^+2^ transients was significantly faster in hiPSC-CMs cultured on aligned nanofibers than in myocytes seeded on standard culture plates (Fig 6B). These results demonstrate that hiPSC-CMs cultured on aligned nanofibers have a faster calcium cycling rate and hence can contract at a higher frequency than cells maintained on a flat surface.

### Electrical properties of hiPSC-CMs seeded on nanofiber

**Fig 7:**
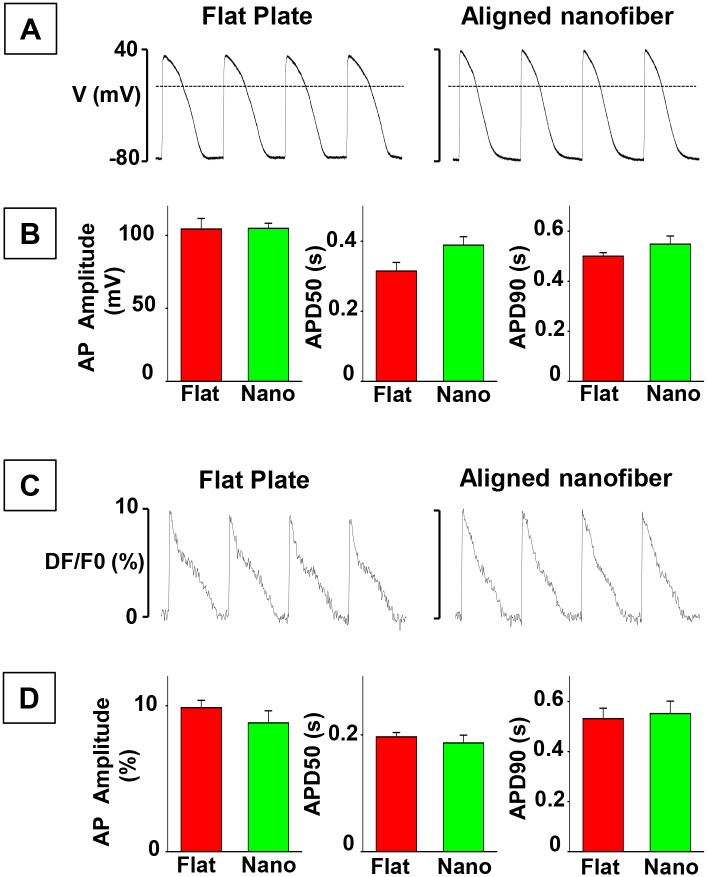
Electrical properties of hiPSC-CMs. **A)** Representative action potential (AP) traces recorded during 1 Hz stimulation in patch-clamped single human iPS-CM seeded on flat bottom or on aligned nanofiber-coated coverslips. Dotted lines indicate 0 mV level. **B)** Summary graphs showing average data for AP amplitude and AP duration at 50 (APD50) and 90 (APD90) % repolarization levels, respectively. Data was recorded in cells from flat plate (Flat, n = 5) and aligned nanofiber (Nano, n = 4) groups, respectively. Resting potential was -74± 2 mV in flat bottom, and -74± 1 mV in aligned nanofiber groups. **C)** Representative action potential (AP) traces recorded in syncytium of human iPS-CM seeded on flat bottom or on aligned nanofiber-coated coverslips. APs were evoked by electrical field stimulation at 1 Hz and were recorded using voltage-sensitive dye di-4-AN(F)EPPTEA. **D)** Summary graphs showing average data for AP amplitude and AP duration. Data was obtained both flat plate (Flat, n = 6) and aligned nanofiber (Nano, n = 6) preparations, respectively.

To compare electrical properties of cells seeded on flat plate and on aligned nanofibers, we used current-clamp mode of patch-clamp technique. hiPSC-CMs were seeded at low density in order to allow membrane voltage recordings from single cell. Action potentials (AP) were induced at 1 Hz frequency. As shown in Fig 7A and 7B, neither the amplitude of AP nor duration was different in hiPSC-CMs plated on flat bottom plate and aligned nanofibers. Of note, the resting potential was also similar (-74± 2 mV in flat plate and -74± 1 mV in aligned nanofiber groups).

We further studied electrical properties of hiPSC-CMs under conditions when cells were seeded at high density to form multiple electrical connections with neighboring cells. To record changes AP from cell syncytium we used voltage-sensitive dye di-4-AN(F)EPPTEA [15]. As illustrated in Fig 7C and 7D, AP properties were similar in hiPSC-CMs seeded on flat-plate and aligned nanofiber groups.

### Synchronized beating of cardiomyocytes

Spontaneous beating of cardiomyocytes on both surfaces was noted within a few days. The beat rate of aligned cardiomyocytes (14.2±0.5 beats/min) was significantly (p<0.05) increased when compared to cardiomyocytes cultured on flat plate (9.5±0.6 beats/min). The beating rate of hiPSC-CMs cultured on the standard flat surface (S1 Movie, **20X**) at 2 weeks was less coordinated than spontaneous contractility of hiPSC-CMs cultured on the aligned nanofiber (S2 Movie, **20X**). At two weeks, a spontaneously contracting layer of hiPSC-CMs on aligned nanofiber shows coordinated, synchronized beating of the entire cardiac patch as seen with light microscopy (S3 Movie, **10X)**.

### Expression of cardiac genes non-failing human heart and hiPSC-CMs

**Fig 8:**
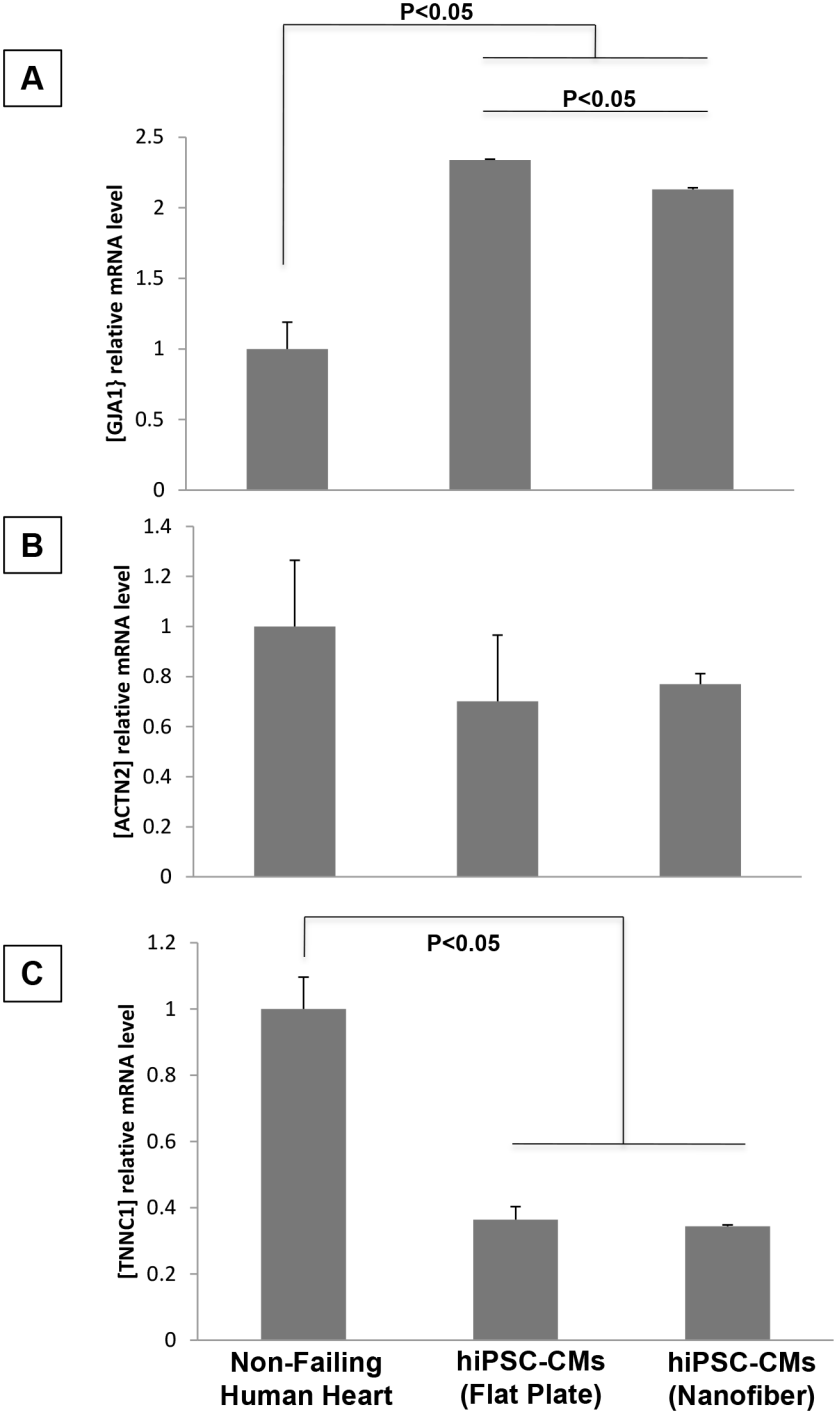
Assessment of cardiac genes by qRT-PCR. mRNA levels of three cardiac markers connexin-43 (GJA1), a-actinin (ACTN2), and troponin-I (TNNC1). **A)** hiPSC-CMs had approximately two fold greater levels of GJA1 mRNA compared to human heart tissue (human 0.78 vs hiPS-CM on flat plates 2.69, n = 6, p = 0.001) **B)** No significant differences in a-actinin mRNA were found between groups **C)** hiPSC-CMs of both groups had approximately 1/3 the amount of troponin-I mRNA compared to non-failing human heart tissue (human 1.06 vs hiPSC-CMs on flat plates 0.38, n-6, p = 0.01).

We have compared mRNA levels of mature cardiac markers (GJA1, ACTN2 and TNNC1) in non-failing human heart tissues with hiPSC-CMs cultured on standard cell culture plates and aligned nanofiber patches. Both groups of hiPSC-CMs had a 2-fold increase in connexin-43 mRNA and a decrease in troponin-C mRNA to about 1/3 compared to non-failing human heart tissues (Fig 8A and 8C). There were no significant differences in alpha-actinin mRNA expression among the three groups (Fig 8B). In contrast, we did not observe any significant differences in mRNA levels of alpha-actinin and troponin-I in hiPSC-CMs cultured on flat cell culture plates versus aligned nanofiber patch.

## Discussion

Replicating the structural properties of the myocardium when fabricating tissue constructs for cardiac tissue engineering, is more likely to promote cardiomyocyte integration and electrical-mechanical coupling [20]. The aligned nanofiber scaffold used in this study demonstrates two potential benefits over culturing techniques on flat cell culture plates. First, the aligned structure allows the tissue constructs to develop anisotropic mechanical properties that mirror native myocardium [18]. Second, the aligned nanofibers guide cardiomyocyte morphology which more closely resembles a normal cardiomyocyte phenotype and possible improved mechanical function. Our study demonstrated the anisotropic orientation and change in human iPSC-derived cardiomyocyte morphology when cultured in an aligned nanofiber environment as compared with a standard culture plate. This monolayer of cells more closely approximates non-failing human heart morphology including the appearance of defined sarcomeres not seen with cells cultured on standard culture plates. The robust expression of actinin and connexin-43 seen in hiPSC-CMs, cultured on both surfaces, provides additional evidence of the maturity and terminal differentiation of these cardiomyocytes. However, of note the organization of cells and thus arrangement of cellular and gap proteins was similar to normal cardiac tissue when cultured on the aligned nanofiber surface. In addition to the structural alignment of hiPSC-CMs on aligned nanofiber, cells cultured on the nanofiber also exhibited more efficient calcium cycling and contractile function. Cardiomyocytes cultured on the nanofiber were noted to have a more rapid calcium cycling rate than cells cultured on standard culture plates. This higher cycling rate enables these cells to contract at a higher frequency than cells maintained on a flat surface.

PLGA is a FDA-approved biodegradable polymer which can be tuned to achieve different degradation times [21,22]. The orientation and morphology of the patch material appears to be an important determinant of patch functionality [23–25]. A recent report indicated that a PLGA micro-grooved thin film seeded with embryonic stem cell derived ventricular cardiomyocytes was readily paced by regular electrical stimulation and resistant to arrhythmogenesis when compared to a non-grooved PLGA film.[26]. The relationship between cardiomyocyte alignment and the nature of their function has been reported by other investigators [27–30]. A study by Woodhouse et al., found that aligned electrospun polyurethane scaffolds led to the anisotropic organization of rod-shaped mESCs-derived cardiomyocytes and improved their sarcomere formation compared with those cultured alone [29]. In another study, Eschenhagen et al. increased the alignment of neonatal rat cardiac myocytes in 3D collagen I matrix by applying a phasic unidirectional stretch for 6 days [27]. Compared with the unaligned cells, the aligned cardiomyocytes exhibited two to four-fold higher contractile forces and a 14–44% decrease in twitch duration under basal conditions and after stimulation with calcium or the β-adrenergic agonist isoprenaline. Most recently, Asiri et al reported alignment of carbon nanofibers in PLGA using voltage improved human cardiomyocyte density compared with human cardiomyocytes cultured on PLGA without carbon nanofibers.[31] The use of carbon nanotubules embedded in a poly(glycerol sebacate) (CNT-PG) scaffold has demonstrated reduced electrical impedance, lower excitation threshold and higher maximum capture rate of the seeded rat cardiomyocytes. Stronger spontaneous synchronized beating was noted with CNT-PG compared with isolated rat cardiomyocytes cultured on PG alone.[32]

Further, our results also demonstrated that hiPSC-CMs seeded on aligned nanofibers exhibited significantly higher beating rates when compared to those seeded on flat plates. This suggests that the proper alignment of cardiomyocytes with neighboring cells may provide more optimal coupling for electrical signal propagation and synchronous cell contractions required for proper cardiac function [28]. On the other hand, the electrical properties measured via patch-clamp technique did not show any significant differences in amplitude nor duration when stimulated at 1 HZ frequency in flat plate versus aligned nanofiber groups.

While other groups have generated a cardiac patch with scaffolding capable of supporting stem cell maturation [33] the novel aspects of our biodegradable cardiac patch are the orientation of human cardiomyocytes on aligned nanofibers mimicking the alignment of cardiomyocytes in the host adult heart. Vacanti et. al. have demonstrated contractile nanofiber grafts, but with no fiber alignment [34] and others have demonstrated the benefits of endothelial cells [35] on neovascularization. The literature is indecisive as to which cell populations provide greater efficacy [36,37]. Recent studies have demonstrated that myocardial scaffolding alone could provide benefits by mechanical support to the heart even in the absence of cells in the scaffold [38,39].

In the current project, we have demonstrated the feasibility of generating a novel biodegradable, aligned nanofiber cardiac patch which facilitates human inducible pluripotent stem-cell derived cardiomyocytes orientation to enable coordinated cardiomyocyte contraction of this patch *in vitro*. Future work is needed to move from a thin layer of functional cardiomyocytes to a thicker multi-cell layer, perhaps incorporating different cell types, to achieve true tissue reengineering and a functional cardiac patch.

## Conclusions

Overall, an aligned nanofiber scaffold seeded with hiPSC-CMs, induces an anisometric response yielding a monolayer of highly developed cardiomyocytes superior to hiPSC-CMs seeded on a flat smooth surface typical of the standard tissue culture plate. The expressed phenotype of hiPSC-CMs on aligned nanofiber more closely replicates mature native cardiomyocytes in terms of mRNA expression, mitochondrial and sarcomere formation, calcium cycling and electro-mechanical conduction.

## Supporting Information

S1 Movie Spontaneous contraction of HiPSC-CMs cultured on standard flat plate at 2 weeks (20 X). (MOV)

S2 Movie Spontaneous contraction of HiPSC-CMs cultured on aligned nanofiber at 2 weeks showed increased coordinated spontaneous contraction compared with hiPSC-CMs cultured on standard flat plate (S1 Movie) (20 X). (MOV)

S3 Movie At two weeks, a spontaneously contracting layer of hiPSC-CMs on aligned nanofiber shows coordinated, synchronized beating of the entire cardiac patch as seen with light microscopy (10X). (MOV)
