# Correction: Evaluation of Changes in Morphology and Function of Human Induced Pluripotent Stem Cell Derived Cardiomyocytes (HiPSC-CMs) Cultured on an Aligned-Nanofiber Cardiac Patch

**DOI:** 10.1371/journal.pone.0141176

**Published:** 2015-10-16

**Authors:** Mahmood Khan, Yanyi Xu, Serena Hua, Jed Johnson, Andriy Belevych, Paul M. L. Janssen, Sandor Gyorke, Jianjun Guan, Mark G. Angelos

The image for Fig 3 is incorrect. Please see the corrected Fig 3 here.

**Fig 3:**
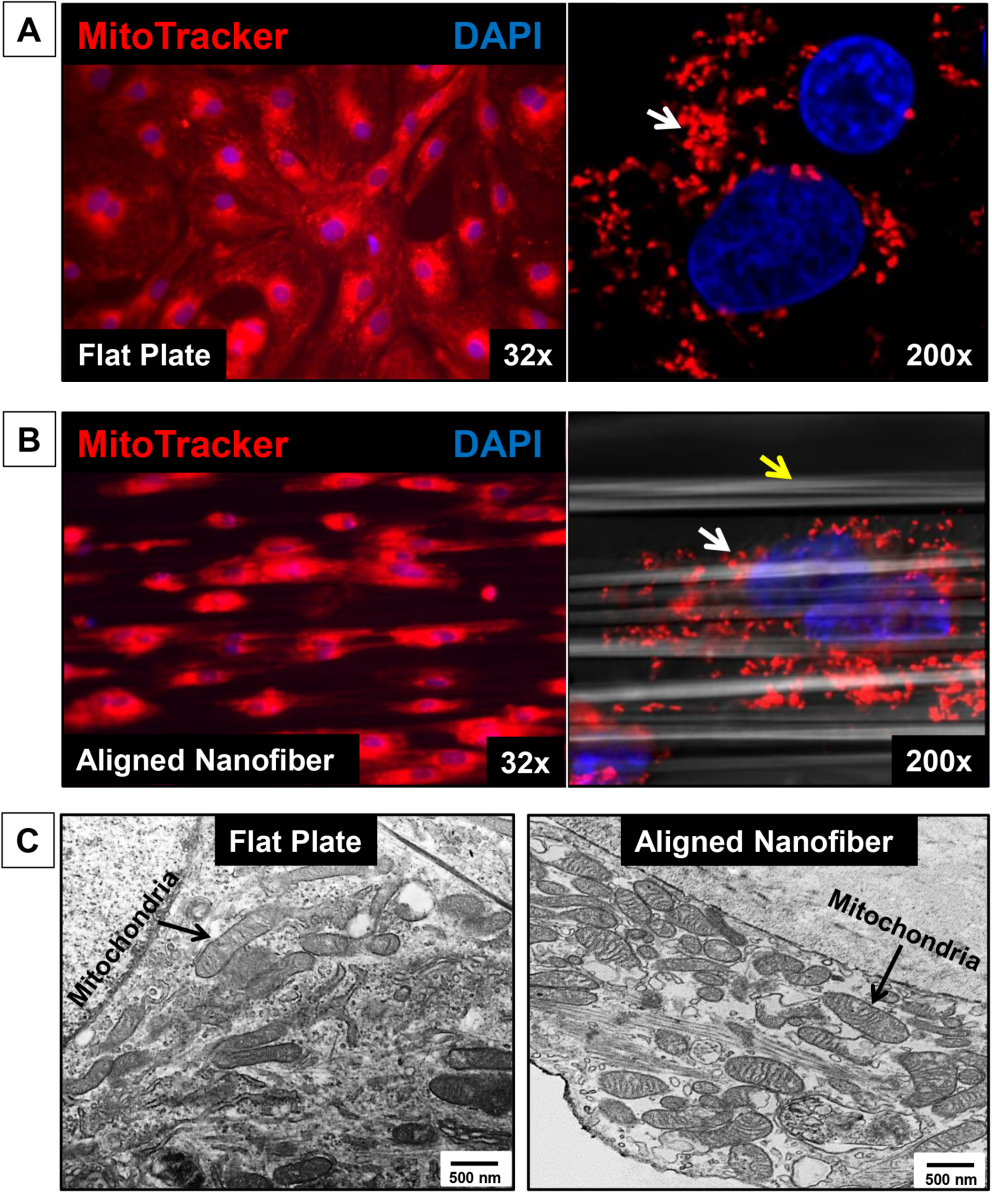
Confocal Imaging of mitochondria. Mito-tracker red staining shows alignment of Human iPSC-Cardiomyocytes seeded on **A)** Flat surface vs **B)** Aligned-nanofiber coated coverslips (32x & 200x). **C)** TEM imaging showing comparison of mitochondrial morphology and arrangement of hiPSC-CMs seeded on flat plate versus aligned nanofiber groups.
